# High-Throughput Phenotyping Approach for Screening Major Carotenoids of Tomato by Handheld Raman Spectroscopy Using Chemometric Methods

**DOI:** 10.3390/s20133723

**Published:** 2020-07-03

**Authors:** Hacer Akpolat, Mark Barineau, Keith A. Jackson, Mehmet Z. Akpolat, David M. Francis, Yu-Ju Chen, Luis E. Rodriguez-Saona

**Affiliations:** 1 Department of Food Science and Technology, The Ohio State University, 110 Parker Food Science and Technology Building, 2015 Fyffe Road, Columbus, OH 43210, USA; akpolat.1@osu.edu (H.A.); chen.6599@buckeyemail.osu.edu (Y.-J.C.); 2 Department of Nutrition and Dietetics, Bayburt University, 69000 Bayburt, Turkey; 3 Lipman Family Farms, 315 E New Market Road, Immokalee, FL 34142, USA; Mark.Barineau@lipmanfamilyfarms.com (M.B.); Keith.Jackson@lipmanfamilyfarms.com (K.A.J.); 4 Informatics Institute, Gazi University, 06500 Ankara, Turkey; zahid.akpolat@gmail.com; 5 Department of Horticulture and Crop Science, The Ohio State University, 119 Williams Hall, 1680 Madison Avenue, Wooster, OH 44691, USA; francis.77@osu.edu

**Keywords:** tomato carotenoids, handheld Raman spectroscopy, chemometrics, artificial neural networks

## Abstract

Our objective was to develop a rapid technique for the non-invasive profiling and quantification of major tomato carotenoids using handheld Raman spectroscopy combined with pattern recognition techniques. A total of 106 samples with varying carotenoid profiles were provided by the Ohio State University Tomato Breeding and Genetics program and Lipman Family Farms (Naples, FL, USA). Non-destructive measurement from the surface of tomatoes was performed by a handheld Raman spectrometer equipped with a 1064 nm excitation laser, and data analysis was performed using soft independent modelling of class analogy (SIMCA)), artificial neural network (ANN), and partial least squares regression (PLSR) for classification and quantification purposes. High-performance liquid chromatography (HPLC) and UV/visible spectrophotometry were used for profiling and quantification of major carotenoids. Seven groups were identified based on their carotenoid profile, and supervised classification by SIMCA and ANN clustered samples with 93% and 100% accuracy based on a validation test data, respectively. All-*trans*-lycopene and β-carotene levels were measured with a UV-visible spectrophotometer, and prediction models were developed using PLSR and ANN. Regression models developed with Raman spectra provided excellent prediction performance by ANN (r_pre_ = 0.9, SEP = 1.1 mg/100 g) and PLSR (r_pre_ = 0.87, SEP = 2.4 mg/100 g) for non-invasive determination of all-*trans*-lycopene in fruits. Although the number of samples were limited for β-carotene quantification, PLSR modeling showed promising results (r_cv_ = 0.99, SECV = 0.28 mg/100 g). Non-destructive evaluation of tomato carotenoids can be useful for tomato breeders as a simple and rapid tool for developing new varieties with novel profiles and for separating orange varieties with distinct carotenoids (high in β-carotene and high in *cis*-lycopene).

## 1. Introduction

Tomato fruit color is one of the most appealing characteristics for consumers in the market [1]. Carotenoid pigments are mainly responsible for the color of the skin and flesh of tomatoes providing yellow, orange, and red colors [2]. There is a growing interest in health benefits of different types of carotenoids because of their health promoting properties such as prevention of certain cancer types and cardiovascular diseases, reduction of age-related macular degeneration and improvement of eye health [3]. These health benefits are usually attributed to antioxidant properties of carotenoids and their interaction with free radicals [4]. Breeding for different tomato colors is a vigorous area of research because of consumer visual appeal and their attributed health benefits [5]. It has been shown that advancements in genetic technology can provide solutions for many specific purposes in plant breeding. However, utilizing the information provided by genetic technology is only possible when it is linked to phenotypic properties of the plant in a real-world setting [6].

High-performance liquid chromatography (HPLC) coupled with diode arrays or mass spectrometry (MS) detectors have been utilized for carotenoid analysis due to high precision, reproducibility and low detection limits [7]. UV-visible spectrophotometry is also extensively utilized for carotenoid analysis using the linear correlation between absorbance and concentration of carotenoids [3,8]. Other methods used less frequently for carotenoid analysis and profiling include matrix-assisted laser desorption ionization time-of-flight spectrometry (MALDI-TOF) [9], supercritical fluid chromatography coupled with mass spectrometry [10], and NMR [11]. However, none of these techniques are suitable for routine and in-field testing due to the cost and nature of the analysis (sample preparation, extraction of carotenoids, risk of degradation, etc.). Non-destructive methods can overcome the drawbacks of standard techniques, since no extraction and sample preparation is involved exposing carotenoids to degradation and/or isomerization, and can be utilized in the field providing true profile and level of the carotenoids [12].

Altering the levels and profiles of certain bioactive and nutritional compounds is possible through naturally occurring genetic variation [1]. However, the cost and labor of conventional methods of measuring these traits have limited characterization for breeding decisions [6]. A rapid and effective phenotyping method for carotenoids in the field would facilitate breeding selections, and tools are becoming possible with the developments of handheld sensors and computational approaches [13]. A rapid and non-destructive method could enhance the development and production of carotenoid-rich commodities to meet consumer demand for healthier foods [14]. 

Vibrational spectroscopy has received increased interest as a promising non-invasive and field-based high-throughput phenotyping platform providing rapid solutions in comparison to labor-intensive, time-consuming, costly, and laboratory-dependent traditional techniques. Infrared spectroscopy was reported by Rubio-Diaz et al. [15] as a rapid and cost-efficient method to profile tomato carotenoids. However, their method was destructive and required extraction of the lipid phase of the tomatoes. Raman spectroscopy is another fingerprinting technique that has shown success for carotenoid analysis, although there are limited reports, including the use of Raman chemical imaging to detect lycopene changes during ripening of tomatoes [7], spatially offset Raman spectroscopy to evaluate internal maturity of tomatoes [16], and resonance Raman spectroscopy for quantification of carotenoid compounds in fruits and vegetables [14]. 

Although handheld Raman spectroscopy has been tested for the detection of tomato ripening [17], it has not been tested before for the classification and quantification of different carotenoid profiles of tomatoes as a non-destructive method. Portable techniques have the advantage of bringing flexibility and rapidity to the location of analysis, which can be utilized by plant breeders, quality control personnel, or purchasing agents. Therefore, we aimed to test the feasibility of handheld Raman spectroscopy to discriminate and quantify the dominant carotenoids in tomatoes as a proof of concept for handheld Raman spectroscopy in the quality analysis of fruits and vegetables.

## 2. Materials and Methods

### 2.1. Tomato Samples

Tomato samples (n = 106) with different carotenoid profiles and colors were obtained from the Ohio State University (Wooster, OH, USA), and Lipman Family Farms (Immokalee, FL, USA). There were 6 different colors of tomatoes with varying carotenoid profiles; red (n = 51), orange (n = 31), yellow (n = 10), green (n = 3), black/brown/purple (n = 7), pink (n = 4). Although the color of tomatoes is somewhat representative of the presence of carotenoids, the carotenoid profiles might be different for samples irrespective of visual coloration. For example, orange tomatoes can be obtained due the presence of high beta-carotene (*beta*), high tetra-cis-lycopene (*tangerine*), or ripening inhibitor alleles (*rin* or *alc* alleles). Similarly, pink and purple coloration in some tomatoes may be due to differences in anthocyanin pigments in the skin of the fruit due to colorless epidermis (*y*) or anthocyanin fruit (*Aft*) alleles. Alternatively, purple may be due to the retention of chlorophyll in green-flesh (*gf*) tomatoes in the presence of trans-lycopene. Finally, the green-flesh gene combined with low carotenoid (*r* allele) result in fruit that are green at maturity. Therefore, assignment of carotenoid profiles and classes was made after HPLC data evaluation using retention times and spectrum of individual pigments.

### 2.2. Raman Spectral Data Acquisition

**Figure 1:**
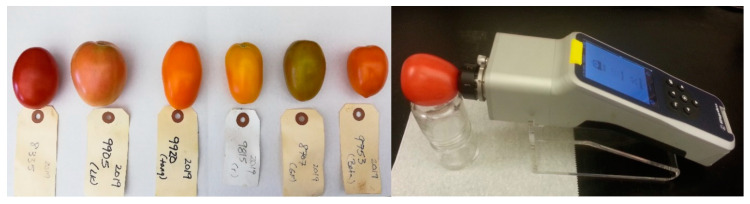
Tomato samples and surface scan using Rigaku’s Progeny handheld Raman spectrometer.

Tomatoes were washed with tap water to remove dirt from the surface and then dried with paper towels. The surface of the intact tomatoes was scanned using a handheld Raman spectrometer, Progeny™ (Rigaku Analytical Devices, Wilmington, MA, USA). The handheld Raman spectrometer featured a 1064-nm excitation laser and thermoelectrically cooled InGaAs 512 pixel detector with a spectral range of 200–2500 cm^−1^ operating at 8 cm^−1^ spectral resolution. The laser power was set at 400 mW and exposure time was 10 s with 30 averages to maximize signal-to-noise ratio without damaging the sample. Three spectra were collected for each sample from three different points on the fruit surface (Figure 1). A background was collected after each sample spectrum collection. 

### 2.3. Profiling Carotenoids by HPLC

After spectral collection, tomatoes were blended using a laboratory blender to be immediately extracted and analyzed by reverse phase high-performance liquid chromatography (HPLC). First, carotenoids were extracted using the method described by Anthon and Barrett [18] with slight modifications. An amount of 8 mL of hexane/ethanol/acetone solvent (HEA) 2:1:1 (v:v:v) was used as extraction solvent for 0.1 g sample. After vortexing for one minute, samples were stored for 20 min in the dark to prevent degradative effects of light and to allow for complete extraction. Following extraction, one ml of deionized water was added to separate phases. The samples were left for 10 more min in dark to allow a complete phase separation. For HPLC analysis, 3 mL of the hexane layer was transferred into amber glass tubes and the organic phase was removed by drying under nitrogen gas. Dried samples were dissolved in 3 mL of methanol/methyl tert-butyl ether (MTB) (1:1/v:v) solvent and filtered using Agilent 0.45-µm PTFE filters into amber HPLC vials. 

Filtrated aliquots (100 µL) were injected via autosampler into an Agilent HPLC (Agilent Technologies Inc., Santa Clara, CA, USA) equipped with a quaternary pump, a diode array detector (DAD), and a degasser. A C_30_ column (5 µ, 250 × 4.6 mm) (YMC Inc., Allentown, PA, USA) was used for separation of different carotenoids during an 80-min run. HPLC conditions described by Rubio-Diaz et al. [15] were used to separate carotenoids. Mobile phases consisted of A: 81% methanol, 15% MTBE, and 4% water and B: 90% MTBD, 7% methanol, and 3% water. A 75 min linear gradient run was used from 0 to 88% B at room temperature with a flow rate of 1.0 mL/min. Carotenoids show different peak shapes and maximum wavelength absorption according to the type of carotenoid in their UV-vis spectra [3]. Therefore, different wavelengths were used for determination of phytoene (290 nm), phytofluene (348 nm), lutein, β-carotene, tetra-*cis*-lycopene, and δ-carotene (450 nm), and all-*trans*-lycopene (470 nm). Tentative identification of carotenoids was performed based on retention times and specific UV-vis absorption spectrum of each compound. The analysis of HPLC chromatograms were made using ChemStation software (Agilent Technologies Inc., Santa Clara, CA, USA). HPLC data were used for class assignments of tomato samples by looking at common patterns in carotenoid profiles. 

### 2.4. Quantification of Carotenoids by UV-Visible Spectrophotometry

Lycopene and β-carotene concentrations in tomatoes were determined using the method described by Anthon and Barrett [18] using the same extraction procedure as described in HPLC analysis section. A Cary 50 UV-vis spectrophotometer (Agilent Technologies Inc., Santa Clara, CA, USA) was used to perform absorbance measurements in triplicates. Concentrations of all-trans-lycopene and β-carotene were calculated using extinction coefficients and absorbance readings. The extinction coefficients used in this study were 172 and 139 mM^−1^, and the maximum absorption wavelengths were 503 and 451 nm for lycopene and β-carotene, respectively. Our all-trans-lycopene dominant samples had many other minor carotenoids as well; however, these minor carotenoids have usually lower absorbance than all-trans-lycopene, so there is minimal interference by those at 503 nm. On the other hand, β-carotene has a maximum absorption wavelength at 451 nm [18]. 

### 2.5. Chemometric Analysis

#### 2.5.1. Partial Least Square Regression (PLSR)

Partial least squares regression (PLSR) is a quantitative technique for developing predictive models by combining features from multiple linear regression and principal component analysis (PCA). Usually it is useful when dependent variables were predicted using a large set of independent variables (predictors). The prediction of dependent variables is achieved by extracting orthogonal factors from predictors to obtain the best predictive power [19]. Since PLSR can analyze highly correlated data and a large number of variables, it is suitable for spectral data analysis [20]. Pirouette software (version 4.0, Infometrix Inc., Woodville, WA, USA) was used to create PLSR models for quantification of lycopene and β-carotene. Average of three spectra was used for each sample, since averaged spectra provided a better performance during model development. All spectral data was mean centered and Savitzky-Golay second derivative (15-point window) and smoothing were used to improve the quality of the spectral signal as they performed best among other pre-processing methods. Leave-one-out approach was used to perform internal cross-validation. Twenty percent of the samples from the whole data set was used as validation set to externally validate the models. Correlation coefficient of cross validation (r_cv_), correlation coefficient of prediction (r_pred_), standard error of cross validation (SECV), and standard error of prediction (SEP) were used to evaluate model performances. High correlation coefficients and low errors show a good prediction model [21]. Outliers were detected using outlier diagnostic tools of the software by evaluating the presence of any large or unusual residuals. 

#### 2.5.2. Soft Independent Modelling of Class Analogy (SIMCA)

Classification algorithms were built in Pirouette software (version 4.0, Infometrix Inc., Woodville, WA, USA) using the SIMCA method. SIMCA is a supervised classification technique, which allows clustering samples based on independent PCA conducted for each class separately [22]. Assignment of classes was based on HPLC data, and SIMCA was used to evaluate the ability of Raman spectroscopy to discriminate between different carotenoid profiles in tomatoes. For each sample, the average of three spectra was used for model development, since averaged spectra provided a better classification performance. All spectral data was mean centered and different data preprocessing techniques were applied to obtain best performances. Finally, standard normal variate (SNV) and Savitzky-Golay second derivative (25-point window) were selected to improve the quality of the spectral signal as they performed best in our dataset. SNV corrects the scattering effect normalizing each spectrum by standard deviation of responses across the entire spectral range [23]. Second derivative removes the non-linear background signal, which might be different for every sample [24]. Model performances were evaluated based on scores plot, discriminating power, and interclass distances (ICD). Scores plot was used for visualization of data showing the first three principle component (PC) and projections of data in a three-dimensional environment. Outlier diagnostics were performed using Mahalanobis distance and sample residual [25]. Discriminating power plot shows which region or wavenumber is most important for the separation of classes. ICD indicates how different classes from each other using residual standard deviations of all variables. A class distance over three suggests well-separated classes [22].

#### 2.5.3. Classification by Artificial Neural Networks (ANN)

Artificial neural networks (ANN) is a nonlinear computational approach employed for classification, clustering, pattern recognition, and quantification purposes [26]. ANN mimics how a biological brain works with a network of neurons processing information and signals from neighboring neurons [27]. Many applications in the literature have been reported using ANN in analysis of various foods, including classification of red wines [28], maturity of dry-cured hams [29], classification of vegetable oils [30], evaluation of olive oil stability [31], classification of olive oils cultivars [32], detection of synthetic colorants [33], geographical traceability of bottled spring water [34], and classification of cow’s milk [35] to name just a few. Advantages of ANN over linear chemometric methods includes its ability of dealing with non-linearity allowing for a better fit of the data, insensitivity to noise providing more accurate prediction, adaptability permitting the model to modify its structure accordingly for the changing environment, and generalization allowing to fit new data to the trained model [26]. 

In our study, ANN approaches were employed for classification and quantification modeling. Multilayer perceptron (MLP) feedforward approach [32] trained with a back-propagation (BP) algorithm was utilized to minimize the error by updating the network weights [28]. To prevent overfitting, we used dropout regularization technique [36] along with early stopping approach. For optimization, adaptive moment estimation (ADAM) was used with default parameters recommended by Kingma and Ba [37]. The neurons in the hidden layer were activated using rectified linear unit (ReLU) activation function, while Softmax function was used for the neurons of the output layer to compute the probability of classes [38]. Loss was calculated with categorical cross entropy (CCE) as an error function to measure the network performance [31]. Variable (spectral) selection was performed using a range scanning approach starting from the first input neuron (first wavelength of spectrum) to the last one with a sliding window (62-point) by moving forward two steps (wavelength) each time. The best spectral region was selected based on the lowest error rate obtained. The average of three spectra from each sample was used, and all spectral data was mean centered. Total number of trained models included different combinations of hyperparameters (i.e., dropout rate from 0 to 1, number of hidden layers from 1 to 2, number of neurons from 2 to 250). Number of epochs for each trial was determined by the early stopping function, which prevents overfitting by stopping training once the model performance stops improving or begins to overfit [39]

We defined the Raman spectral data as “inputs” while the “output” consisted of the classes of carotenoids for classification or the concentration of the specific carotenoid for quantification. The data set was divided into a training set, a validation set for assessing the overfitting, and a test set for assessing the performance of the ANN model [34]. The samples were randomly divided into training:validation:test for ANN model using a 80%:10%:10% structure for classification and a 60%:20%:20% for quantification. 

ANN model development was performed in Python using Keras (an open source modular neural network library).

## 3. Results

### 3.1. Characterization of the Carotenoid Profiles of Tomatoes

**Figure 2:**
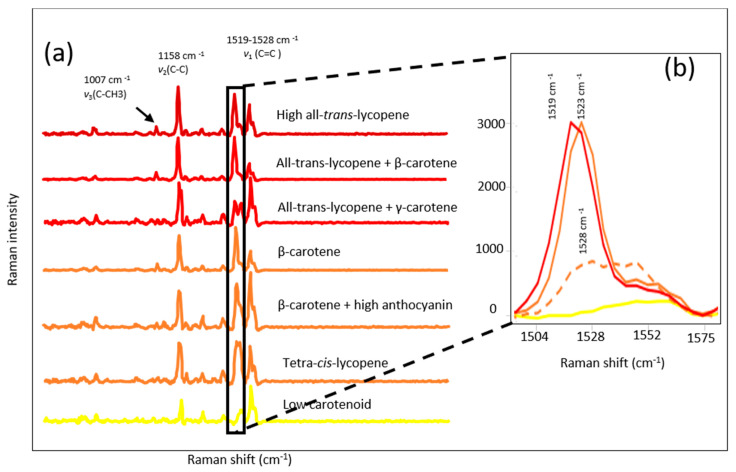
Raman spectra of different carotenoid profile tomatoes (**a**); colors are representative of fruit color with a few exceptions such as brown/black samples in “β-carotene + high anthocyanin group”, and ν1 band shapes and locations for different class of tomatoes (**b**); Red = all-*trans*-lycopene samples, solid orange = β-carotene, dashed orange= tetra-*cis*-lycopene, yellow= low carotenoid samples.

The Raman spectra of selected red, tangerine, orange (high Beta-carotene), and yellow (low carotenoid) tomato breeding material are shown in Figure 2a. All Raman profiles showed common bands at 1007 cm^−1^ and 1158 cm^−1^ associated with C-CH_3_ in-plane-rocking and C-C stretching, respectively. However, visual evaluation of Raman spectrum showed a shift at around roughly 1520 cm^−1^ corresponding to C=C stretching depending on the major carotenoid of the tomato (Figure 2b). Tomatoes containing predominantly all-*trans*-lycopene showed a band maximum at 1519 cm^−1^, tomatoes having tetra-*cis*-lycopene and β-carotene showed a shift in the band centered at 1523 and 1528 cm^−1^, respectively. The low carotenoid samples did not show any significant carotenoid band in the Raman spectrum (Figure 2b). The position of the C=C stretching Raman band was inversely related to the length of the conjugated polyene chain, isomerization (*trans* vs. *cis*), the side groups of polyene chain and the presence of other constituents bonded to carotenoids in the plant material [13,40,41,42]. 

**Figure 3:**
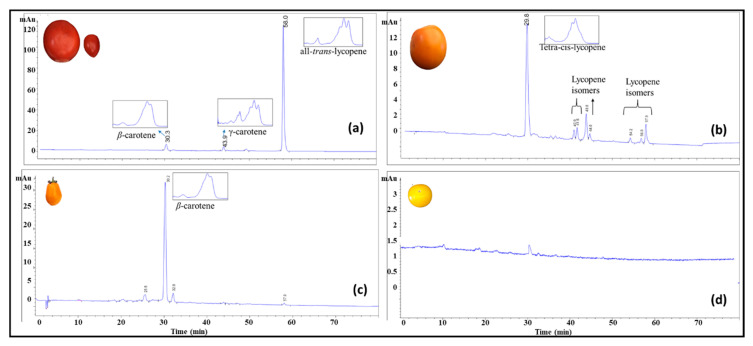
High-performance liquid chromatography (HPLC) chromatogram monitored at 450 nm and their corresponding absorption spectrum collected by the photodiode array detector for carotenoids in selected tomato breeding material. (**a**) Red tomatoes; (**b**) tangerine tomatoes high in tetra-cis-lycopene; (**c**) tangerine tomatoes high in β-carotene; (**d**) yellow tomatoes low in all carotenoid.

High performance chromatography was used for carotenoid profiling of the tomato breeding material including yellow, orange, red, and purple coloration. Four main groups were identified based on HPLC data including (1) all-*trans*-lycopene, (2) β-carotene, (3) tetra-*cis*-lycopene, and (4) low carotenoid samples carrying the *r* gene (Figure 3). 

**Figure 4:**
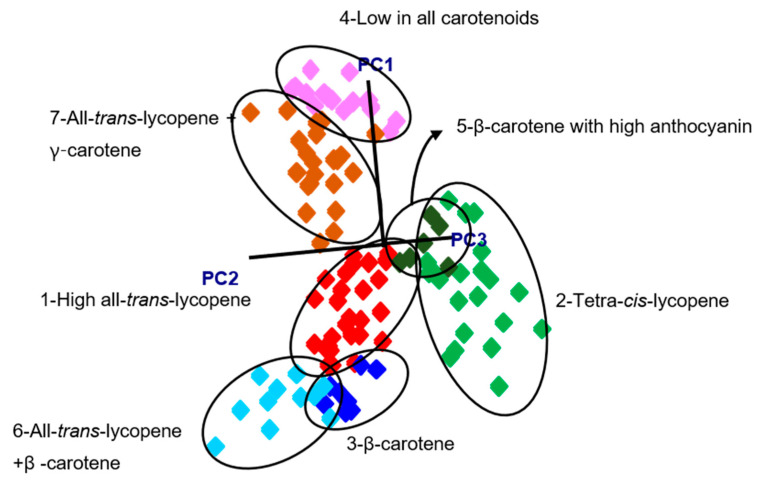
Soft independent modelling of class analogy (SIMCA) 3D class projections using the information in the 1370–1670 cm^−1^ region.

After the assignment of classes based on HPLC carotenoid profiles, pattern recognition algorithms were built using SIMCA. The spectra were preprocessed before analysis by using standard normal variate (SNV) and Savitzky-Golay second derivative filtering (25-point window) to remove scattering noise effects and correct for baseline drift. The SIMCA model was able to separate the groups based on the information in the 1370–1700 cm^−1^ region and revealed seven clusters instead of the four suggested by HPLC. These seven different groups were based on their dominant carotenoid profile as well as the presence of other carotenoids or anthocyanins resulting in the following classes: (1) high levels of all-*trans*-lycopene and no β-carotene, (2) all-*trans*-lycopene and β-carotene, (3) all-*trans*-lycopene and γ-carotene, (4) β-carotene (and low lycopene), (5) β-carotene and anthocyanins, (6) tetra-*cis*-lycopene, and (7) low in all carotenoids. The class projections (Figure 4) of the seven groups showed well separated classes in a three-dimensional environment using the first three principal components (PC). Interclass distances (ICD) values ranged from 3.4 to 38.5. The highest ICD (38.5) was between low carotenoid and the all-trans-lycopene + β-carotene group, while lowest ICD (3.4) was between tetra-*cis*-lycopene and the all-*trans*-lycopene (high) group. Our study showed that all ICD values between classes were >3 (Table 1) with significant separation of clusters [22]. 

The discriminating power graph in the SIMCA model shows the wavelengths mainly responsible for the separation of the groups [22], which can be representative of specific chemical structures. The band at 1523 cm^−1^ was responsible for separation of different groups in the discriminating power graph. This explains that the wavelength shift caused by predominant carotenoids of tomatoes was mainly responsible for separation, since 1523 cm^−1^ corresponds to the area for the ν_1_ band at around 1519–1530 cm^−1^ depending on the carotenoid type. We validated our classification model using a separate data set (20% of the whole data set) to evaluate prediction performance. The external validation revealed that the SIMCA classification model had a 93% accuracy for the prediction of independent samples.

Additionally, we investigated ANN as a non-linear method for classification of tomato carotenoids based on handheld Raman spectroscopy using the class assignments from SIMCA algorithms. ANN models using the full spectrum were not able to separate classes very well, so variable selection was important for the predictive performance by eliminating uninformative variables and noise [43]. The range from 1377 to 1671 cm^−1^ gave the lowest error and highest accuracy and was used as input (n = 60) for the network. This range was very similar to the range used in SIMCA analysis and included the band around 1520 cm^−1^ corresponding to C = C stretching band shift of carotenoids. 

Among 1000 trials to find the optimum hyperparameters of the model, 100% accuracy was reached for 35 different ANN models. The highest accuracy (100%) and minimum test loss (0.03804) was obtained with a 0.4 dropout rate using 242 neurons in one hidden layer. The eight most efficient networks were selected based on the highest accuracy obtained for the test set and the lowest test loss (Table 2). The properties were given in Table 2 arranged in a decreasing order of test loss. Test loss was calculated with CCE loss function; the lower the test loss means the higher the probability for a particular class assignment [44]. The number of epochs shown in the table was quite different for each model because of the early stopping function, which stops the training process once the network performance starts deteriorating to prevent overfitting. Similar classification abilities were obtained with different network topologies and hyperparameters. 

### 3.2. Quantification of All-Trans-Lycopene and β-Carotene by PLSR and ANN

**Figure 5:**
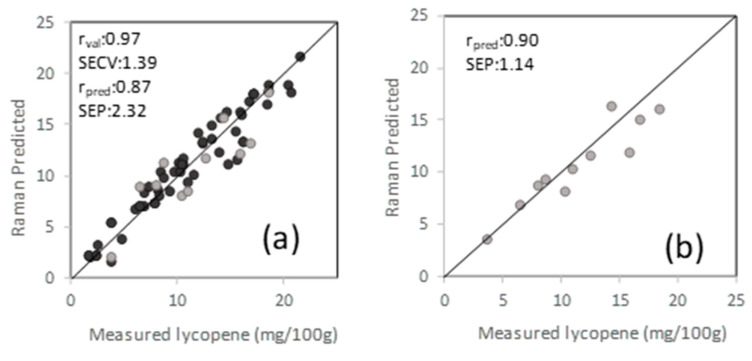
Regression model for all-trans-lycopene quantification for PLS (**a**) and artificial neural network (ANN); (**b**) black representing calibration set, and gray representing external validation test set.

Among 106 samples, 58 had all-*trans*-lycopene, and 17 had β-carotene as the dominant carotenoid based on HPLC results. Quantification of all-*trans*-lycopene and β-carotene was performed using UV-Vis spectrophotometer, and concentration ranged from 1.8 to 21.5 mg/100 g for all-*trans*-lycopene and 1.6 to 9.5 mg/100 g for β-carotene. PLSR was used to correlate spectral and reference analysis data using the Savitzky-Golay second derivative filtering with a window size of 15 and smoothing that removed the effect of variation in the baseline [45]. PLSR models were generated using a calibration (80%) and external validation (20%) data sets. Cross-validation (leave-one-out) of the calibration set selected four latent variables that explained most of the variance in the data and avoided overfitting, giving a correlation coefficient of and standard error of cross-validation of 1.4 mg lycopene/100 g (Figure 5). By using the external validation set, the PLSR model gave prediction performances of 0.87 for correlation coefficient and a standard error of prediction (SEP) of 2.3 mg lycopene/100 g sample. The only report to our knowledge available for lycopene quantification in tomatoes using a handheld Raman spectrometer (1064 nm) has showed an R^2^ of 0.88 in their regression model, with no error value or external validation data set reported [46]. Sheng et al., [47] reported on a non-destructive method for lycopene determination in cherry tomatoes, with a lycopene concentration of 7.51 ± 1.27 mg/100 g, using portable NIR spectroscopy, reporting a correlation coefficient of 0.80 and SEP of 0.74 mg/100 g. Our data set had a wider lycopene range and comprised different types of tomatoes, including round, Roma, cherry, and grape tomatoes.

The PLSR prediction algorithms built for β-carotene were also promising with r_cv_ of 0.99 and SECV of 0.28; however, the number of samples available was limited (n = 17). Therefore, further investigation is necessary with more samples. 

The feedforward MLP ANN was used to correlate Raman spectral data and carotenoid reference values to predict all-*trans*-lycopene content of tomatoes. Since the number of β-carotene samples was very limited, an ANN model was not developed for β-carotene. Data was divided into three groups; training (60%), validation (20%), and test (20%) sets. The same spectral region (1377–1671 cm^−1^) was used as in the classification model. Dropout regularization technique, ADAM optimization technique was used as described in the previous sections. ReLU activation function was used in both hidden and output layers. The best MLP model and optimum hyperparameters of the model were obtained with trial and error. The best performing ANN model included 219 of neurons in a hidden layer with a dropout rate of 0.5 and an epoch cycle of 1000. Regression coefficient for test data was found as 0.90 with an RMSE value of 1.14 mg/100 g for quantification of all-*trans*-lycopene. 

## 4. Conclusions

Handheld Raman spectroscopy combined with different pattern recognition techniques allowed for the non-destructive classification of carotenoid profiles in tomatoes based on dominant carotenoid types. Raman spectroscopy allows for time-efficient (less than 5 min), user-friendly, and in-field analysis of carotenoids and our green approach allows carotenoids to be analyzed in their natural environment avoiding the risk of isomerization and degradation during the extraction process. Classification of tomatoes based on their dominant carotenoid profile were promising by using SIMCA and ANN pattern recognition techniques. SIMCA showed a classification accuracy of 93% when an independent data set was tested for validation of the model, while ANN provided 100% classification accuracy in the test data set. The same spectral information was used for quantification of all-*trans*-lycopene by using PLSR and ANN modeling giving good regression coefficients > 0.9 and standard error of prediction (SEP) of 2.3 and 1.1 mg/100 g for PLSR and ANN, respectively. The improved predictive performance of ANN could be attributed to its ability to deal with non-linear functions. 

Our findings fill the gap in the literature for a non-destructive and portable technique for profiling and quantifying major tomato carotenoids utilizing the strong laser penetration ability of Raman spectroscopy for in-situ and non-destructive fruit analysis. The technology can provide breeders real-time information in improving tomato varieties based on unique carotenoid profiles and colors.

## Figures and Tables

**Table 1:** SIMCA inter-class distances among four groups.

	All-*Trans*-Lycopene (High)	Tetra-*Cis*-Lycopene	β-Carotene	Low Carotenoids	β-Carotene + High Anthocyanin	All-*Trans*-Lycopene + β-Carotene	All-*Trans*-Lycopene (Low)
all-*trans*-lycopene (high)	0.0	3.4	6.1	18.4	5.7	7.5	6.2
tetra-*cis*-lycopene		0.0	6.9	8.7	4.7	10.7	7.1
β-carotene			0.0	34.9	12.9	3.8	19.5
low carotenoids				0.0	9.9	38.5	5.4
β-carotene with high anthocyanin					0.0	17.0	6.3
all-*trans*-lycopene + β-carotene						0.0	20.9
all-*trans*-lycopene (low)							0.0

**Table 2:** The most efficient 8 networks selected from 2000 networks based on classification accuracy and test loss.

Network Topology *	Dropout	Epoch	Classification Accuracy (%)	Test loss (CCE)
60-242-7	0.4	814	100	0.03804
60-178-7	0.1	738	100	0.05484
60-240-7	1	746	100	0.06449
60-106-7	1	1417	100	0.06616
60-199-7	0.4	852	100	0.07257
60-226-7	0.1	499	100	0.07568
60-201-7	0.7	1388	100	0.07896
60-238-7	0.4	712	100	0.089558

* number of neurons in input layer, hidden layer, and output layer, respectively.
